# Influence of Charge Regulation on the Performance of Shock Electrodialysis

**DOI:** 10.1021/acs.iecr.2c03874

**Published:** 2023-02-07

**Authors:** Harm T.M. Wiegerinck, Reinder Kersten, Jeffery A. Wood

**Affiliations:** Soft Matter, Fluidics and Interfaces, MESA+ Institute for Nanotechnology, University of Twente, 7500AE Enschede, The Netherlands

## Abstract

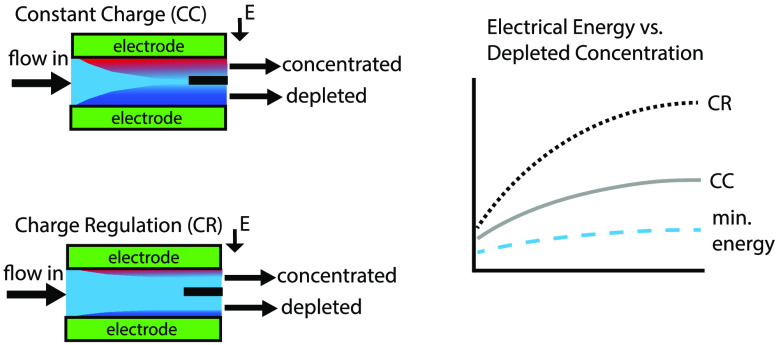

In order to understand the ion transport in a continuous cross-flow shock electrodialysis process better, numerous theoretical studies have been carried out. One major assumption involved in these models has been that of a constant surface charge. In this work, we considered the influence of charge regulation, caused by changes in salt concentration, on the performance of a shock electrodialysis cell. Our results show that, by including charge regulation, much higher potentials need to be applied to reach the same degree of desalination, compared to the constant surface charge model. Furthermore, we found that operating at higher potentials could lead to substantial Joule heating and therefore temperature increases. Although somewhat lower potentials were required in the nonisothermal case versus the isothermal case with charge regulation, the required energy input for desalination is still much higher than the thermodynamic minimum. This works highlights the important role charge regulation can play in a shock electrodialysis process.

## 1 Introduction

**Figure 1:**
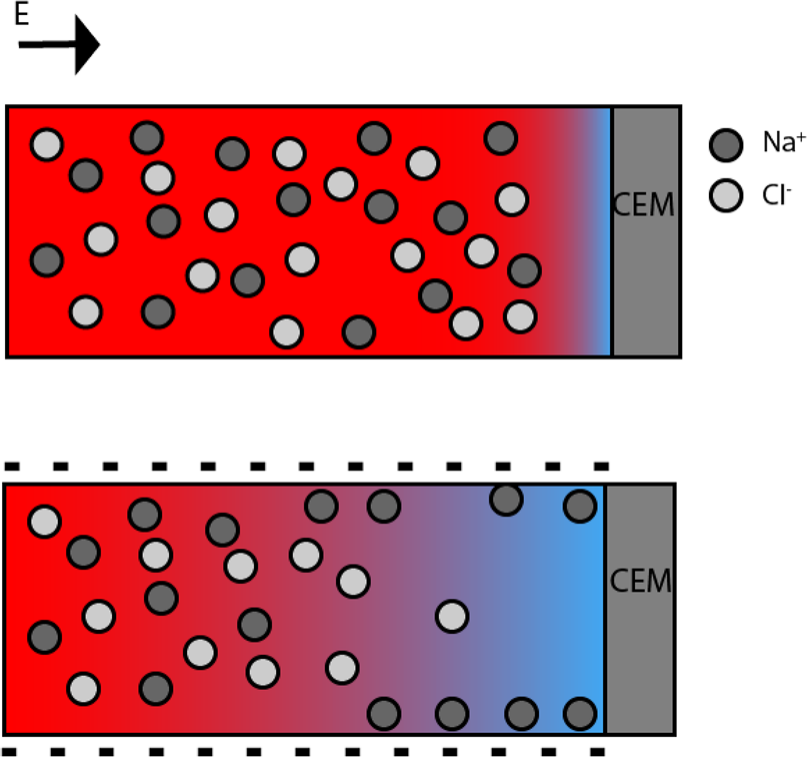
(Top) Schematic of depletion layer in free electrolyte connected to a cation exchange membrane when potential is applied. (Bottom) Schematic representation of depletion layer in charged pore connected to a cation exchange membrane. Red color represents a high salt concentration, and blue color represents a depleted solution. CEM = cation exchange membrane. The figure represents the same geometry shown in refs (7) and (10).

Shock electrodialysis (shock ED) is a relatively new electro-driven separation technique that has been studied at laboratory scale to remove salt from saline waters, such as brackish water^1,2^ and seawater,^3^ as well as the removal of radionuclides from nuclear plant cooling water^4^ and the removal of lead^5^ and other heavy metals from waters.^6^ Shock ED works by applying an electric field over a charged porous medium. In the past, it was found that by applying an electric field over a microchannel, connected to an ion selective nanochannel,^7−9^ a large polarization layer depleted of salt (depletion layer) forms on the order of several millimeters (see Figure 1). This principle is the basis for the shock ED process, where a porous medium is contacted with an ion exchange membrane.^1,10^ The transport mechanism of ions is briefly explained: in water-filled charged porous media, such as silica networks, there is a fixed surface charge of dissociated silanol groups present that is screened by a layer of counterions at the silica/water interface (see the bottom of Figure 1). When an electric field is applied to a free solution near a cation exchange membrane (see the top of Figure 1), this leads to cation movement from left to right. Since the cations can move through the cation exchange membrane and anions move away from the membrane, after some time, the region very close to the membrane will be depleted of ions. In free solution, ions can only transport through the depletion layer by diffusion, which leads to a relatively thin depletion layer. In contrast, in a negatively charged pore of the porous medium (see the bottom of Figure 1), the transport through the depleted region is possible for cations by electromigration (surface conduction), which can be much faster than diffusion. This mechanism results in a much larger depletion layer near the membrane, compared to the free solution case.

**Figure 2:**
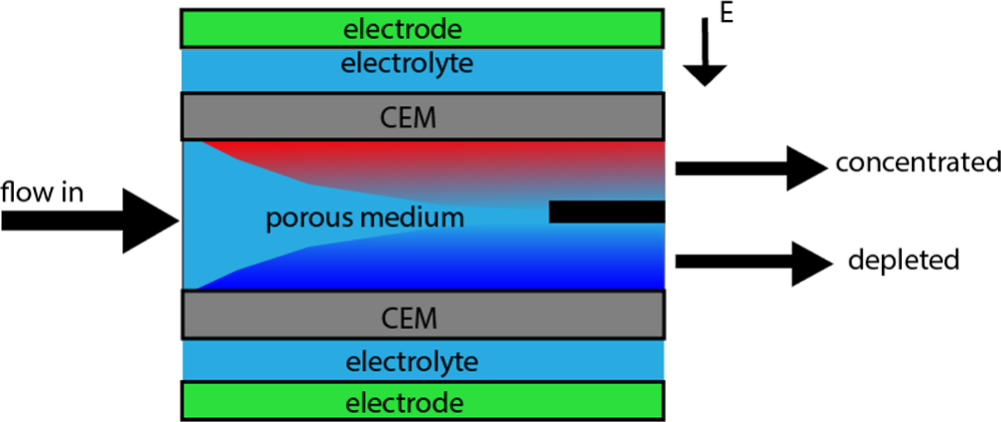
Schematic representation of the shock electrodialysis (shock ED) setup. The figure represents the same geometry shown in ref (11).

The type of ion exchange membrane that is needed for the shock ED process depends on the charge of the counterions that can transfer near the surface of the pore wall. In the case of a negatively charged porous medium, such as silica, with positively charged counterions, the membrane needs to facilitate the transport of cations through the membrane. Therefore, a cation exchange membrane (CEM) is shown in Figures 1 and 2.^7^

To use the shock ED principle for a continuous process, a cross-flow setup was devised by Schlumpberger et al.^11^ (see Figure 2), where the depleted concentration is separated from the concentrated stream using a splitter. The potential advantage of shock ED, compared to conventional electrodialysis, is that, in addition to desalination, particles and bacteria present in the water can be filtered out in a single unit operation,^12^ which could reduce the total capital cost of the process to produce drinking water from saline water sources.

In order to gain insight into the ion transport, besides experimental shock ED studies,^2−5,12^ several different shock ED models have been developed.^11,13−15^ The first model describing the continuous shock ED process was developed in 2021 by Schlumpberger et al.^11^ This model describes the ion transport based on the Nernst–Planck framework with electroneutrality. Besides this model, more-complex models based on the Nernst–Planck-Poisson framework have been used, which, for example, included protons and hydroxides based on the water equilibrium.^14,15^ While simulations based on these models help in gaining a better understanding of ion transport in the shock ED process, these models all assumed a porous medium with a constant surface charge.

However, it is commonly known that, in reality, almost all materials (silica in particular) have a surface charge that is, to some degree, dependent on both solution pH and salt concentration,^16^ which is known as charge regulation. Therefore, this work will extend the shock ED model used by Schlumpberger et al.^11^ by fully implementing the charge regulation model of Behrens and Grier^17^ and examining the influence of charge regulation on the resulting desalination performance.

When we implemented the charge regulation in the shock ED model, we found that the applied electrical potential required to reach an equivalent desalination degree is substantially higher, compared to that found when assuming a constant surface charge. This raised the question of whether Joule heating and the associated increase in temperature could possibly have a significant impact on the ion transport. Previous studies in our group showed that temperature changes and temperature gradients can have a large impact on ion transport in electrically driven ion transport processes.^18,19^ Consequently, we also considered the effect of Joule heating on the ion transport in the shock ED process through numerical simulations. Finally, we also examined the influence of charge regulation and Joule heating on the resulting energy requirements for operating a shock electrodialysis process using brackish feedwater.

## 2 Charge Regulation Theory

It is commonly known that the surface of silica contains silanol groups that are either charged or neutral and follows the following equilibrium reaction (at acidic pH):^20^
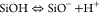
1Consequently, the number of charged silanol groups depends on the local proton (hydronium ion) concentration near the silica surface, which depends on the bulk pH, as well as the salt concentration. In order to get a better understanding on how the salt concentration affects the local proton concentration, we have to take a closer look at the electrostatic double layer near the charged porous medium. According to the Gouy–Chapman theory, the surface charge on the porous medium is screened by a diffuse double layer of primarily counterions and can be approximated as follows as a Boltzmann distribution:
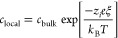
2where *c* is the concentration of ions either locally near the silica surface or in the bulk, *z*_*i*_ the valence of the ion considered,*e* the elementary charge, ξ the interaction potential (zeta potential), *k*_B_ the Boltzmann constant, and *T* the temperature.

When the salt concentration is reduced, there are less ions in the bulk that can screen the porous medium, so the electrostatic interaction potential increases. Consequently, relatively more protons (as well as other cations) are attracted toward the silica surface, which shifts the equilibrium reaction relatively more toward the neutral silanol groups. This means that, even under the assumption that the bulk pH remains constant, the surface charge can change considerably due to salt concentration changes only. Throughout this work, the (bulk) pH is, to remain consistent with the work of Schlumpberger et al.,^11^ fixed at a pH of 7.^20,21^

To included the above-described effect of charge regulation, we used the 1 – p*K*_a_ Stern model to calculate the surface charge of the porous medium, while using the parameters of Behrens and Grier.^17^ Hereafter, we will refer to this model as the Behrens and Grier model.
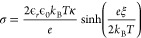
3
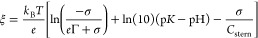
4where
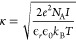
5

Charge density is calculated from the surface charge by using the expression
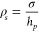
6where κ is the inverse Debye length (the Debye parameter), *e* is the elementary charge, *N*_A_ is Avogadro’s number, *I* is the total ionic strength of the bulk solution, which also accounts for the fixed amount of protons and hydroxides in this work, ϵ_*r*_ is the relative dielectric constant of water, ϵ_0_ is the permittivity in vacuum, *k*_B_ is the Boltzmann constant,*T* is the temperature (Kelvin), σ is the fixed surface charge, ξ is the zeta potential, Γ is the total number of surface sites on silica, p*K* is the logarithmic proton adsorption equilibrium constant for silica, pH is the pH of the solution, and *C*_Stern_ is the Stern capacitance. All these and other important parameters used in the model presented in section 3 are summarized in Table 1 and are consistent with the work of Schlumpberger et al.^11^

A first potential limitation of the model of Behrens and Grier is the validity of the Grahame equation (eq 3), since it is only valid for flat plates and geometries that can be approached as a flat plate due to limited curvature, with respect to the Debye length. For most salt concentrations found within the modeled shock ED system, the average pore size (2.7 μm) is much larger than the Debye length, which is equal to ∼3 nm for a salt concentration of 10 mol/m^3^. Therefore, it suffices to disregard the curvature in the porous medium and treat it as a flat plate geometry. At the lowest depleted salt concentration physically possible (2 × 10^–4^mol/m^3^), the characteristic parameter κ*h*_*p*_ is, at this concentration, ∼0.3, which indicates that the double layer is not necessarily negligible, relative to the average pore size everywhere in this system.

A second potential limitation of the model is that the model only considers the charge density of the mobile ions from the diffuse layer, while also the ions in the Stern layer could, under some conditions, contribute significantly to the transport of ions through the porous medium. However, as evaluated by Leroy et al.,^22^ it was shown that, at a salt concentration of 1 mM, the conductivity of the diffuse layer is already higher, relative to the Stern layer, and the diffuse layer becomes even more dominant with decreasing salt concentration. Therefore, although the inclusion of the Stern layer in the model could change results at relatively high concentrations and at low applied potentials, when a significant depletion layer has formed, it is believed that neglecting the ion transport through the Stern layer is appropriate for the conditions considered here.

As a final note it has to be mentioned that, while the zeta potential is calculated separately in the presented charge regulation model, initially, the zeta potential was fixed at −70 mV, to focus only on the effect of charge regulation on the surface charge density. Later, in section 6.8, we will show that the effect of varying zeta potential is minor compared to the effect of charge regulation on the charge density and the subsequent influence this has on the performance of a shock ED process.

## 3 Model Description

For our simulations, we used the dimensional set of equations of the so-called “cross-flow model” by Schlumpberger et al.,^11^ combined with an electroneutrality condition to describe the ion transport, and fluid flow is described by Darcy flow including electro-osmosis:
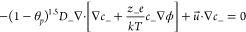
7
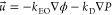
8
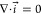
9
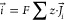
10
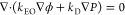
11
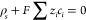
12where
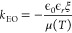
13
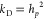
14

The porous medium is modeled as a continuum medium with a fixed porosity and single pore size, instead of a pore size distribution. The diffusion coefficient of ions is estimated using the Bruggeman model. This is a very simple approach to model a porous medium; for example, Schmuck et al.^23^ and Tian et al.^14^ considered more-complex cases. However, by using our simple porous medium approach, we can compare our results, to some extent, with the results obtained by Schlumpberger et al.^11^

Apart from a more-complex porous medium model, assuming a single pore size for the shock ED setup instead of a pore size distribution could possibly have a large impact on the ion transport, since smaller pore sizes lead to an increase of the charge density and subsequently increase the shock ED performance (see Figure S2 in the Supporting Information). However, the opposite is true for larger pores. The model results presented are appropriate for a porous medium with a modest spread in pore sizes.

To consider nonisothermal effects, temperature was determined by solving the energy balance with a Joule heating term for the electrically generated heat:
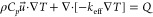
15
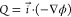
16
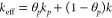
17

A list of the symbols/parameters used in this set of equations is given in Table 1.

**Table 1:** All Relevant Parameters of the Shock ED Model with Values (Where Appropriate) and Units

parameter	symbol	value	reference
diffusion coefficient at 20 °C	*D*_*i*_	Na^+^: 1.33 × 10^–9^ m^2^/s	–
		Cl^–^: 2.03 × 10^–9^ m^2^/s	
ion valency	*z*_*i*_	Na^+^: 1	–
		Cl^–^: −1	
Boltzmann constant	*k*_B_	1.38 × 10^–23^ J/K	–
elementary charge	*e*	1.609 × 10^–19^ C	–
electro-osmotic permeability	*k*_EO_	4.8631 × 10^–8^ m^2^/(V s)	(11)
Darcy permeability	*k*_D_	7.2311 × 10^–14^ m^2^	(11)
surface charge density	ρ_*S*_	–74 375 C/m^3^ or variable	(11)
Faraday constant	F	96485 s A/mol	–
permittivity in vacuum	ϵ_0_	8.85 × 10^–12^ F/m	–
relative permittivity	ϵ_*r*_	78 or variable	–
zeta potential	ξ	–70 × 10^–3^ V	(11)
fluid viscosity	η	10^–3^ Pa s	–
characteristic pore size	*h*_*p*_	2.69 × 10^–7^ m	(11)
inverse Debye length	κ	variable (m^–1^)	–
Avogadro’s number	*N*_A_	6.022 × 10^23^ mol^–1^	–
ionic strength	*I*	variable (mol/m^3^)	–
surface charge	σ	variable (C/m^2^)	–
silanol surface site density	Γ	8 × 10^18^ nm^–1^	(17)
p*K*	–	7.5	(17)
pH	–	7	–
Stern capacitance	*C*_Stern_	2.9 F/m^2^	(17)
fluid density	ρ	998 kg/m^3^	Comsol
fluid heat capacity	*C*_*p*_	silica: 703 J/(kg K)	Comsol
		water: variable	
fluid velocity	*u*	variable (m/s)	-
effective heat conductivity	*k*_eff_	variable	Comsol
heat production	*Q*	variable (W/m^3^)	–
current density	*i*	variable (A/m^2^)	–
applied potential	ϕ	variable (V)	–
volume fraction of particles	θ	0.48	(11)
heat conductivity	*k*_*i*_	silica: 1.38 W/(m^2^ K)	Comsol
		water: variable	
**Commonly Used Abbreviations Specific for This Work**			
ion flux	*J*_*i*_	variable (mol/m^2^/s)	–
electrodialysis	ED		
constant charge	CC		
charge regulation	CR		
electro-osmotic flow	EOF		

## 4 Simulation Details

The simulations were performed with the finite element solver Comsol, and all of the equations are solved with linear shape functions.

**Figure 3:**
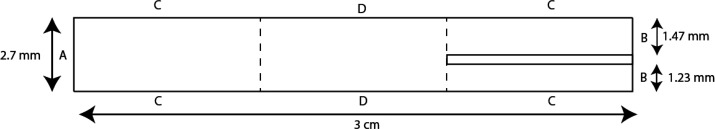
Schematic representation of the simulation geometry, indicating the different types of boundary conditions imposed on the edges and the (approximate) dimensions of the geometry. Splitter is not to scale.

In Figure 3, the simulation domain is schematically represented with all of its types of boundary conditions labeled by letters. This domain only consists of the porous medium part without the membrane and electrolyte channels (see Figure 2). Furthermore, the domain is split in three different domains, where only, as described in detail below, in the second domain an electrical potential is applied at the boundaries. In the current simulations, boundaries D can be considered as a perfect cation exchange membrane.

At boundary A, a fixed inlet pressure (1.1 bar) is applied, to operate under constant pressure conditions; also, the concentration is fixed to the inlet concentration. At boundary B, the pressure is set to 1 bar and the ions are only transported by convection. Boundaries C are modeled as impermeable walls for all ions. The electrical potential is set at the top boundary and the lower D boundary acts as a ground. To ensure that a solution is found for the simulations, the applied potential is slowly increased by a parametric sweep starting at 0.01 V until 10 V for the constant charge model, until reaching 300 V for the charge regulation model and 90 V for the nonisothermal charge regulation models. Since only the anion equations are solved for and the cation distribution is calculated via the electroneutrality condition, no anions can pass boundaries D by the no-flux condition, which results in the net transport of cations over these boundaries.

The splitter in this work is modeled, contrary to the basic model in the literature,^11^ as an actual splitter domain with a very small width (0.02% of the height, 0.0054 mm), where the model equations were not applied to, which makes the splitter domain impermeable to both ions and fluid flow. This is in contrast to the original cross-flow model, where a no-flux condition was imposed on a line to mimic the splitter.

For the nonisothermal charge regulation simulations, at boundary A, the fluid comes in at a fixed temperature of 20 °C, and at boundary B, the heat is transported by convection. All the other boundaries (C and D) were made thermally insulating, which means that the maximum impact from Joule heating was assessed in these simulations.

To test whether the numerical solutions of the simulations were mesh independent, an arbitrary starting mesh was refined by a factor of 2 and the average current density at the upper boundary D of the refined mesh was compared to the current density of the old starting mesh for all the applied potentials for which the model was solved. We continued to refine the mesh in this way until the relative difference in the overall current density between the old and new mesh was <0.5%. The final mesh that is used in all simulations and contains 80 rectangular elements in the along the height of the domain and 720 rectangular elements along the length of the domain. The mesh becomes gradually 4 times smaller near all its boundaries, relative to the middle of the domains.

The two-dimensional (2D) profiles were obtained by exporting the results out of Comsol plots. The quantitative results were obtained by averaging the current density at both the membrane boundaries and by averaging the flowrate, anion concentration, and temperature at the fluid flow inlet, or depleted and enriched outlet flow boundaries. All the simulations were performed considering a feed of sodium chloride (NaCl) solution at 10 mol/m^3^ (10 mM), with a constant pH of 7. The inlet pressure was kept fixed at a pressure of 1.1 bar over the length of 3 cm.

## 5 Shock ED Cases

Since we will compare different variations on the basic cross-flow model, in this section, we will discuss all the different models used in this paper. The most simple model is the constant surface charge model, indicated from this point forward as the constant charge (CC) model (*T* = 20 °C). This model is made for comparison purposes, since it is almost identical to the model made by Schlumpberger et al.,^11^ with some minor modifications, as discussed in the previous section to model it properly into Comsol. In this model, the surface charge density of the porous medium is calculated with the charge regulation model of Behrens and Grier,^17^ based on a concentration of 10 mol/m^3^(inlet concentration).

The charge regulation model is modified by implementing the same charge regulation model. However, here, the local concentrations are used to calculate the surface charge density of the porous medium. This model will be indicated from this point forward as the isothermal charge-regulation (CR) model (*T* = 20 °C).

Next to implementing the charge regulation, temperature effects as a result of Joule heating will also be considered in the third model (CR). In this nonisothermal model, three different temperature effects will be included: the ion diffusivity, the viscosity of the water, and the temperature dependence of the proton adsorption on the silica surface sites. In addition to these effects, some other minor temperature effects were explored, such as buoyancy-driven flow and Soret (thermodiffusion) effects; these analyses are provided in the Supporting Information (section S1).

The temperature dependence of the ion diffusivity is calculated based on the Stokes–Einstein relation, while assuming the hydrodynamic radii of the ions will remain constant over the temperature changes obtained in the simulations as an approximation. This is not completely valid for the chloride ion: From 20 °C to 80 °C, its hydrated radius decreases by ∼10%.^24^ Since this effect is only moderate, the Stokes–Einstein relation suffices for this study. This yields the following expression for the temperature dependent diffusion coefficients:
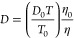
18where η_0_ is the viscosity of water at 20 °C and η is the viscosity at the local temperature. For the viscosity of the water, the built-in temperature dependence in Comsol is used.

Finally the proton adsorption (the p*K*_a_ value) is affected by temperature. With increasing temperature, the protons obtain a higher kinetic energy, which makes them less likely to adsorb onto the silica surface sites. Therefore, a higher temperature leads to a lower p*K*_a_ value and a higher surface charge density of the porous medium. The temperature dependence of the p*K*_a_ is calculated by^25^
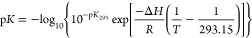
19where Δ*H* is the enthalpy of proton adsorption. For the value of this enthalpy, several different values are mentioned in the literature, from 15 kJ/mol to 90 kJ/mol,^25^ and, more recently, a enthalpy value of 81 kJ/mol was estimated to be a realistic temperature dependence for the silica sites.^26^ In order to capture the maximum effect of the temperature-dependent proton adsorption, a value of 90 kJ/mol was used.

As mentioned in section 3 in all of the previous models, the zeta potential was fixed at −70 mV. In section 6.8, the effect of the zeta potential on the simulation result will be evaluated in more detail, by extending the CR model.

## 6 Results

### 6.1 Concentration, Velocity, and Temperature Profiles

**Figure 4:**
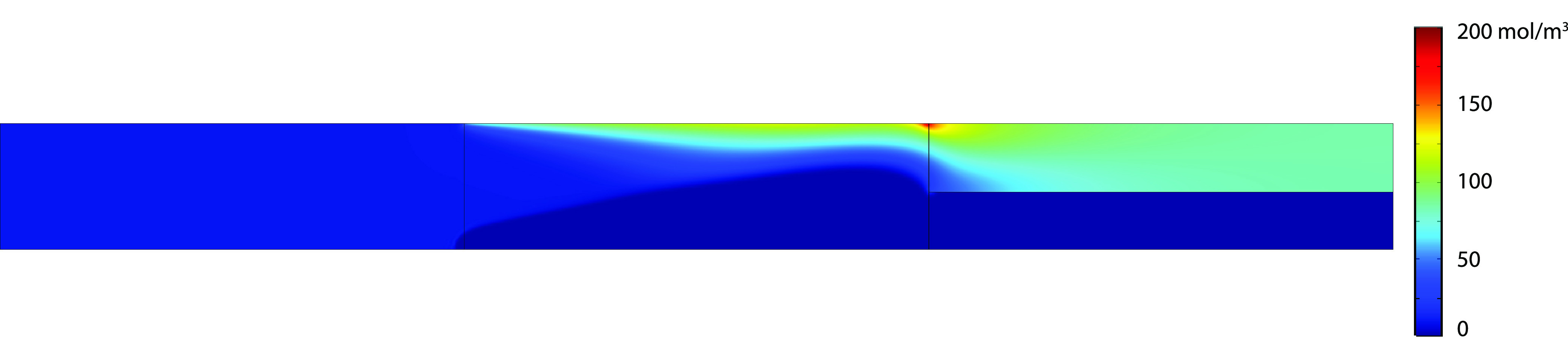
Spatial 2D anion concentration profile of the nonisothermal charge regulation model at an applied potential of 90 V.

**Figure 5:**
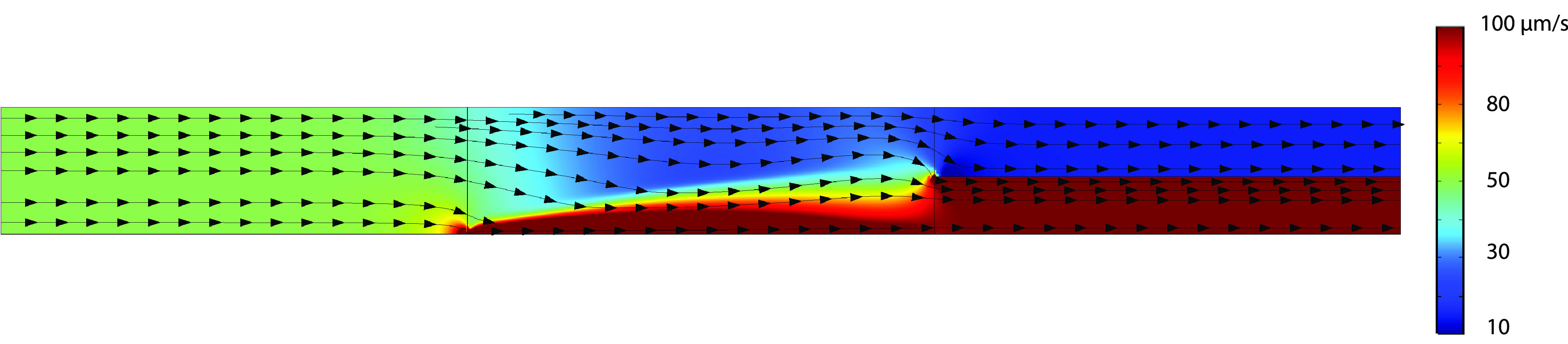
Spatial 2D velocity profile of the nonisothermal charge regulation model at an applied potential of 90 V. Arrows indicate the local flow direction.

**Figure 6:**
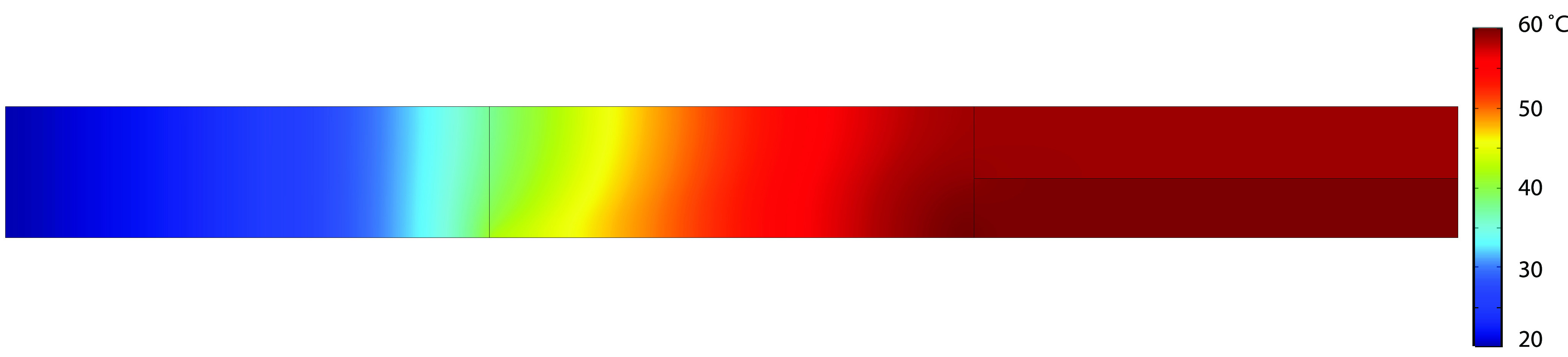
Spatial 2D temperature profile of the nonisothermal charge regulation model at an applied potential of 90 V.

Since the 2D results of the concentration and pressure profile for the constant charge case (CC) was already given in Schlumpberger et al.^11^ and the 2D plots of the isothermal charge regulation provides limited insight, we decided to only show some example profiles of the nonisothermal charge regulation model at an applied potential of 90 V, since this shows the most interesting profiles. The 2D plots for the nonisothermal charge regulation model are given for the anion concentration, the fluid velocity, and the temperature plots (see Figure 4, 5 and 6, respectively). A potential of 90 V is extraordinarily high, especially considering that it is only applied over a few millimeters. In practice, this could lead to other effects neglected in our simulation, such as excessive water-splitting reactions in the porous silica frit,^27^ which prevents the system from complete deionization. However, to be able to compare the different shock ED cases at similar desalination degrees, we decided to show the results up to these high applied potentials.

Figure 4 shows that the concentration depletion ”shock” evolves similar to the shocks presented in the work of Schlumpberger et al.^11^ Another figure that gives some nice insight is the velocity plot, as seen in Figure 5. In this figure, in the first domain essentially, the velocity profile is completely uniform. When the fluid in the second domain, where the electric potential is applied, is dragged toward the bottom due to electro-osmotic flow (EOF). However, because the fluid cannot pass through the bottom boundary, this leads to a higher fluid pressure near the bottom wall, which counteracts the electro-osmotic flow, to some degree. This leads to more horizontal flow lines near the splitter. Because of EOF, fluid is transferred from the concentrated salt stream toward the depleted region, which leads relatively more fluid exiting the depleted outlet, when sufficiently high potentials are applied.

Most of the heat is produced within the region depleted of salt, since the electrical resistance is the highest there and therewith the production of heat. However, the temperature profile (see Figure 6) is quite uniform over the entire height. This suggests that the heat transfer is much faster compared to the mass transport. This can be confirmed by evaluating the Lewis number, which gives the relation between mass diffusivity and heat transport.^28^ By evaluating the Lewis number, we found that the heat transfer through the porous medium is always between ∼30 to 70 times faster than mass transport at low and high temperature, respectively. Therefore, due to the much-faster heat transport, the temperature increases essentially only along the length of the setup, which has implications for the temperature of recovered water if such high potentials would be required.

### 6.2 Surface Charge Density of Silica

**Figure 7:**
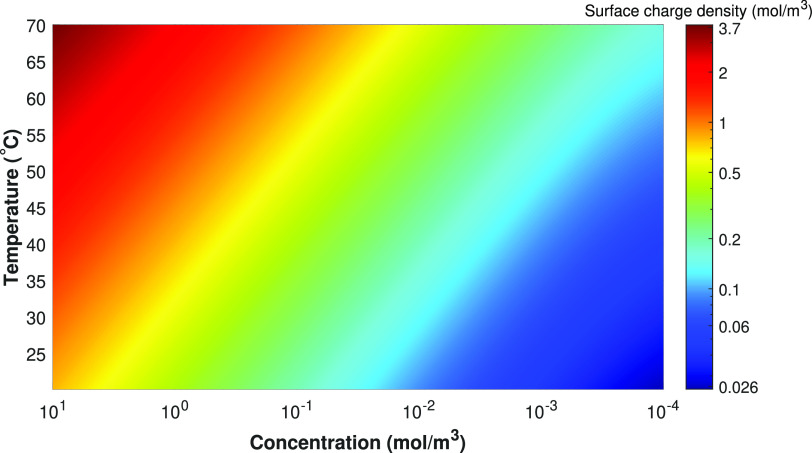
Surface charge density of the porous silica medium, as a function of the salt concentration and temperature. The colors are based on a logarithmic scale.

To give some insight into how the surface charge density will change locally as a function of the local salt concentration and temperature, the charge regulation is solved as function of both these variables, as can be seen in Figure 7.

In Figure 7, it can be seen that the surface charge density decreases roughly 2 orders of magnitude, when comparing the high concentration regime with the lowest concentrations. From this result, it is to be expected that the ion transport behavior of the shock ED process including (nonisothermal) charge regulation can be remarkably different, compared to the constant surface charge density assumption on the porous medium. As can be seen in Figure 7, the effect of temperature on the charge density was not as pronounced as the effect of salt concentration within the range of relevant conditions. From this figure, it is expected that, within the depletion zone, the fixed surface charge density will decrease significantly with concentration.

### 6.3 Salt Concentration versus Applied Potential

**Figure 8:**
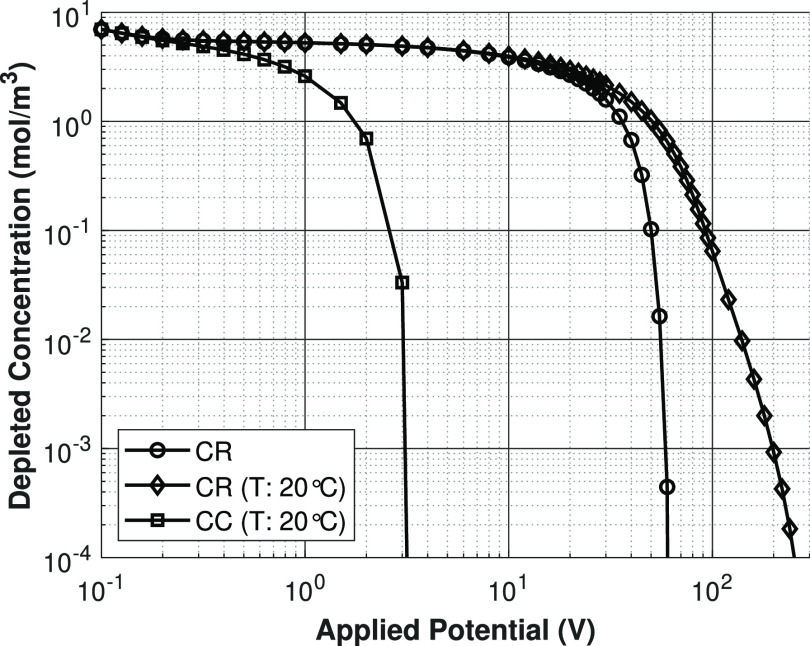
Depleted salt concentration at the outlet versus the applied potential for constant charge case and charge regulation cases. Lines are present for visualization purposes only.

In Figure 8, the depleted concentration at the outlet of the shock ED domain is plotted against the applied potential for a feed concentration of 10 mol/m^3^ NaCl. At low potentials, the behavior of all the models is identical. This means that the behavior at low potentials is completely controlled by the migration of ions, which is proportional to the diffusion coefficient of the ions. Since these are equal for all the models in this regime, the effect of Joule heating at this stage is negligible. Near an applied potential of 1 V, the constant charge case starts to deplete much faster, compared to the charge regulation models. This is caused by the difference in surface charge density, when the depletion layer starts to grow, the electrical resistance is determined by how many ions are still present in the depleted zone. Since the surface charge density decreases quickly with decreasing concentration (see Figure 7), a much lower driving force is needed for a constant-charge porous medium situation, compared to a charge-regulating porous medium, to achieve the same ion flux.

**Figure 9:**
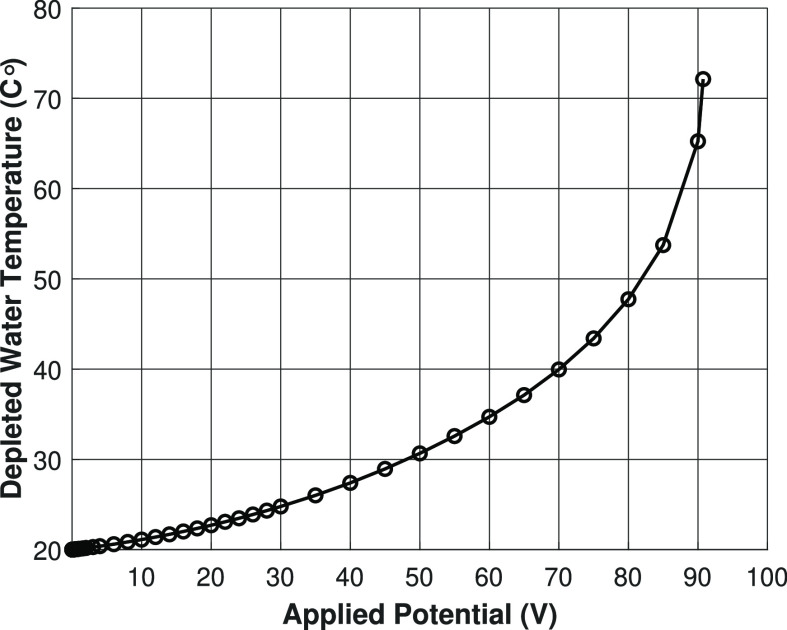
Temperature of the depleted outlet stream versus the applied potential for the nonisothermal charge regulation model. Lines are present for visualization purposes only.

Both the isothermal charge regulation and nonisothermal charge regulation model show identical behavior until ∼40 V. The reason for this result is that the water must be sufficiently depleted of ions, before Joule heating increases the temperature of the salt solution significantly. The point where both the charge regulation models start to deviate coincides with the point where the temperature increases (see Figure 9). Because of both an increased diffusivity of the ions and an increased surface charge density due to decreased proton adsorption on the porous medium, the nonisothermal charge regulation model predicts that the solution can achieve high depletion at lower driving forces, compared to the isothermal charge regulation model.

The implementation of the nonisothermal charge regulation model is set up to show the maximum implication of temperature on the charge regulation. However, in reality, also some heat will leak away via the membrane boundaries and setup walls, which will shift the nonisothermal curve more toward the results of the charge regulation model. Generally, the parameters used in the charge regulation model can also vary from reality, which could change the exact result of the curve. However, due to the general trend of decreasing charge with decreasing salt concentration, it is not expected that the results of the charge regulation model will dramatically affect the difference between constant charge and charge regulation models for a porous medium made of silica.

The temperature for the nonisothermal charge regulation model increases by a quadratic function with increasing applied potential (see Figure 9). This follows from the general heat balance equation, which leads to an equation that is quadratic in the applied potential:
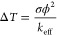
20where σ is the conductivity of the solution, ϕ is the applied potential, and *k*_eff_ is the effective thermal conductivity.

### 6.4 Conductance

**Figure 10:**
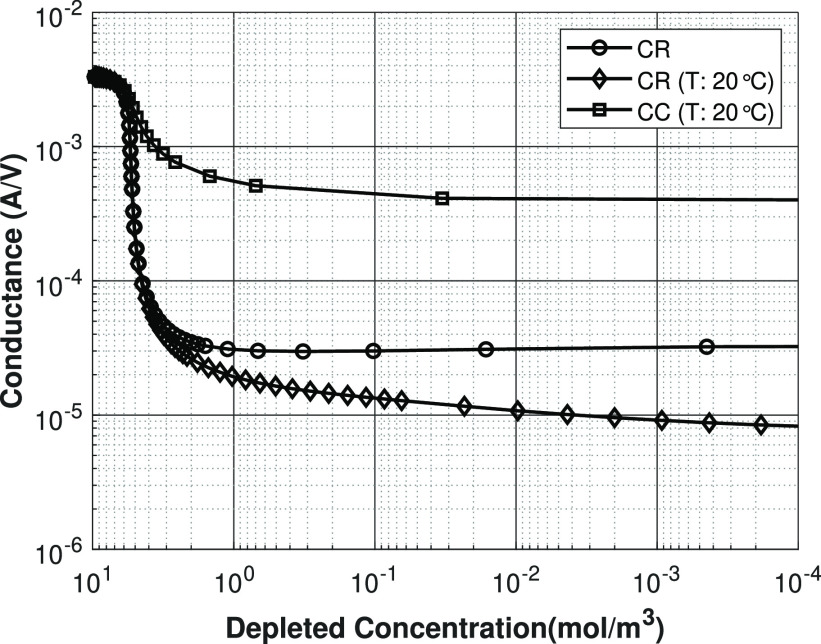
Conductance plotted against the depleted outlet concentration. Lines are present for visualization purposes only.

To get some more insight into the mechanisms that play a role in the ion transport during shock ED, the conductance is plotted against the depleted outlet concentration (see Figure 10). The conductance is calculated by dividing the current density at the electrodes by the applied potential. At depleted outlet concentrations close to the feed concentration (minimal desalination), the conductance has a constant value, which indicates that the process operates in the so-called ohmic regime, where the rate at which the ions transport are transported is directly proportional to the applied electric potential. Furthermore, in this regime, the ion transport is independent of the surface charge density of the porous medium. As the average concentration decreases, the depletion layer forms and increases in size, which results in a sharp decrease of the conductance with average outlet concentration. For the constant charge case, this decrease is only ∼10 times the initial value, because there remains relatively large amount of surface charge on the porous medium, that allows the transport of cations through the depletion layer, while the anions are increasingly excluded from the depletion zone. Because of the surface conduction mechanism, the conductance eventually stabilizes to a plateau at low concentration, because the surface conduction ion transport mechanism is governed by how much surface charge is on the porous medium. Obviously, for a constant charge case, the charge density remains fixed; therefore, based on that, the conductance is expected to show a plateau. In contrast, for the charge-regulating cases, the conductance decrease much more, compared to the initial conductance value, because the charge reduces with salt concentration. In addition, there is no real conductance plateau observed, due to the concentration dependence of the surface charge density. The conductance plateau was also found experimentally in ion transport studies through nanochannels with constant surface charge on its walls.^29,30^ In a follow-up study of Taghipoor et al., it was also shown numerically that when charge regulation effects are taken into account, the conductance does not show a real plateau with decreasing salt concentration.^31^ This is consistent with both the results obtained in experimental studies that are compared by Taghipoor et al. and our current result, that the conductance for the charge-regulation model is not reaching a plateau (see Figure 10).

Interestingly, the nonisothermal charge regulation model shows, in contrast with the isothermal charge regulation model, an almost-constant conductance plateau. This is caused by the diffusion coefficients increasing with increasing temperature and this compensates for the decrease in the surface charge density due to depletion. Additionally, due to the temperature increase a higher surface charge density is obtained compared to the isothermal charge regulation case which results in a higher conductance.

### 6.5 Electro-osmotic Flow

**Figure 11:**
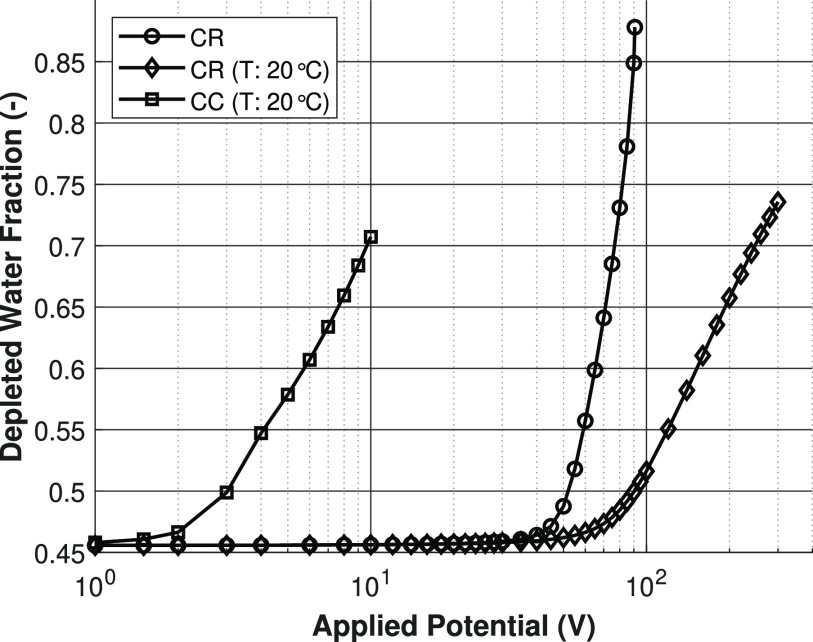
Relative depleted outlet flow rate, with respect to the inlet flow rate versus applied potential. Lines are present for visualization purposes only.

**Figure 12:**
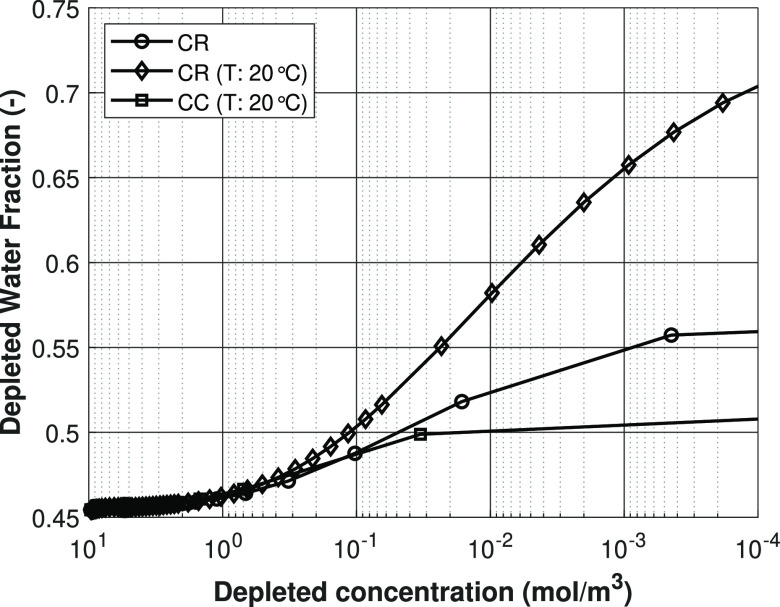
Relative depleted outlet flow rate, with respect to the inlet flow rate versus depleted outlet concentration.

Because of electro-osmotic flow, fluid is displaced from the concentrated zone toward the depleted outlet. In shock ED, electro-osmotic flow has an influence on the recovered water flow when there is a sufficient electric potential drop over the depleted region, which can be seen in Figures 11 and 12. Here, the depleted water fraction is defined as follows:
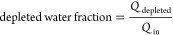
21where *Q*_depleted_ is the flow rate at the depleted outlet (lower boundary B in Figure 3) and *Q*_in_ is the inlet flow rate (boundary A in Figure 3).

In Figure 11, it seems that, for the nonisothermal charge regulation model, the depleted water recovery can be increased until almost 90% of the inlet water exits via the depleted outlet. This would be a remarkable result, since this could mean that, independent of splitter geometry and height, almost all the feedwater can be desalinated. However, for the highest applied potentials, the depleted concentration becomes unrealistically low due to the numerical solver, but the system still assumes the physics of the physical minimum concentration.

This is nicely illustrated when comparing Figure 11 to the result in Figure 12. In these figures, it can be seen that, even when the depleted outlet stream is essentially fully desalinated, the maximum depleted water fraction is only ∼0.6. This means that, in order to have high depleted water recoveries, the applied potential must be increased much more than is actually required to desalinate the brackish water stream completely. Therefore, the increase in water recovery due to electro-osmotic flow could be quite limited under normal desalination conditions.

### 6.6 Comparison with Literature Results

To compare our results with the results presented in Schlumpberger et al.,^11^ we nondimensionalize the current by using the relation
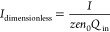
22where *I* is the current obtained from the current density, *z* the ion valency, *n*_0_ the inlet concentration (given as the number of ions per cubic meter), and *Q*_in_ is the inlet flow rate in cubic meter per second.

**Figure 13:**
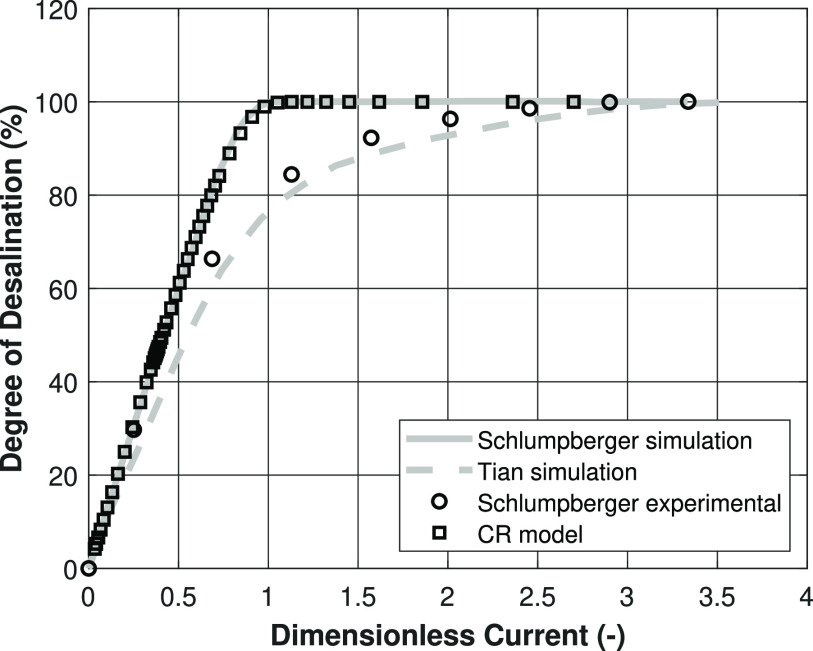
Degree of desalination against the dimensionless current, according to eq 22. Lines are present for visualization purposes only.

In Figure 13, the degree of deionization is plotted against the dimensionless current for the constant charge simulation and experimental results of Schlumpberger et al.,^11^ together with our nonisothermal charge regulation model. For these results, again, 10 mol/m^3^ NaCl is used as feed solution. As can be seen, both simulation results coincide almost perfectly, while the deviation with the experiments at higher dimensionless currents is substantial. One big difference between the performed experiments and the simulations is the presence of protons and hydroxides. Including them into the simulation would reduce the current efficiency of the system, since, next to cations, protons also can be transported through the membrane. For this reason, the model of Tian et al.^14^ aligns quite well with the experimental results, since, in that model, proton and hydroxide transport has been taken into account. However, this figure also shows that making comparisons based on the dimensionless currents can be less insightful, since the Schlumpberger model (equivalent to the CC case in this work) and the CR case, which showed completely different behavior in the preceding results, align quite well in this figure.

**Figure 14:**
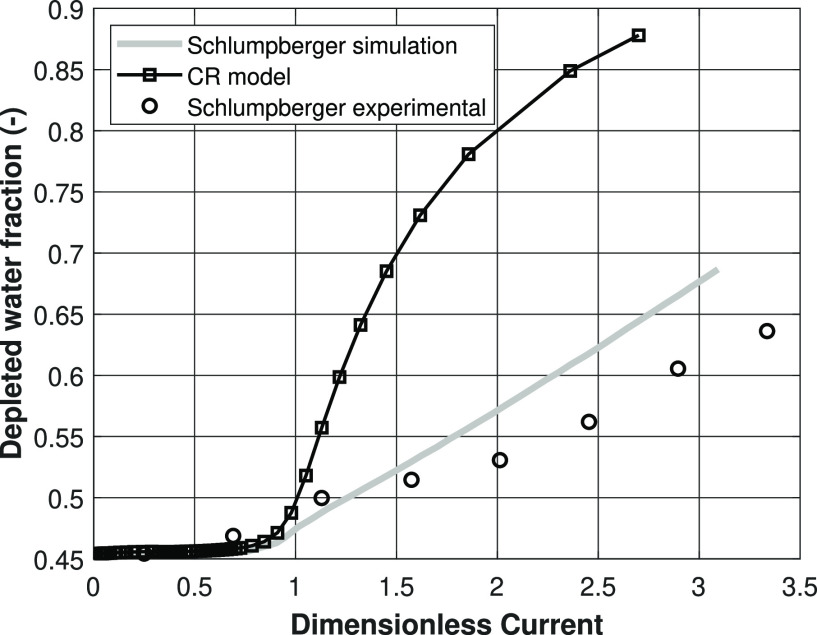
Depleted water fraction against the dimensionless current according to eq 22 for the CR model, simulation results from the literature, and experimental results from the literature. Lines are present for visualization purposes only.

Second, the depleted water fraction is compared to the literature results in Figure 14. The charge regulation models are compared to the both the experimental model as well as the CC model results of Schlumpberger et al.^11^ Clearly, our nonisothermal CR model predict an even higher water recovery, compared to the results of Schlumpberger et al.^11^ This is caused by the fact that a much higher driving force is needed to deplete the solution, so, as a consequence of that, the electro-osmotic water transport will have a much larger contribution at the same dimensionless current. When comparing the simulation results to the experimental work of Schlumpberger et al.,^11^ it shows that the CC model actually seems to be more accurate in describing the experimental results on the depletion water fraction versus the dimensionless current, compared to our CR model.

First of all, this could be caused by not including the protons and hydroxide species into the model. However, this can never be the only reason for the deviations, since, experimentally, the onset of the EOF occurs at much smaller degrees of desalination, compared to simulations, as can be seen by comparing this result with the previous result in Figure 13. Alternatively the deviation between simulations and experiments could be caused by the still simplistic way the electro-osmotic flow is implemented, since it is modeled with the Helmholtz–Smoluchowski equation. In section 6.8, we will check the effect of the EOF on the shock ED performance, based on double layer overlap proposed by Chen et al.^32^ Unfortunately, we could not solve the shock ED model with the more realistic EOF model for sufficiently high applied potentials to show its effect on the depleted water fraction, because, as was shown previously in Figure 12, effective water transport only occurs when the solution is almost completely free of ions. Since we could not solve the simulation up until this regime, we are unable to compare this more-advanced EOF model to the presented experimental results of Schlumpberger et al.^11^

Alternatively, the electro-omotic flow could be completely different due to the inhomogenous nature of the silica frit, which most likely has pores of various sizes, varying widely from the assumed average pore size and porosity, which would make it challenging to find a continuum model that can accurately describe the electro-osmotic flow for an arbitrary porous medium. Nevertheless, as was already shown earlier, the enhanced water recovery becomes only relevant when the salt content in the depleted water stream is negligible. Therefore, it is not the most important result for which an accurate electro-osmotic model is required.

### 6.7 Relevance of Other Effects and Assumptions

In the main results, we included several temperature effects next to the effect of charge regulation. Here, we will briefly comment on some other effects that may also affect the performance but were not covered in the main results section. A more elaborate discussion on these effects can be found in the Supporting Information (sections S1–S3). In those sections, we performed theoretical evaluations with regard to what extent ion transport could be affected by natural convection, thermophoresis, the effect of the temperature dependence of the dielectric constant, and the effect of electro-osmotic vortices. Furthermore, in that section, we comment on our assumption to keep the pH constant. In short, we concluded that, when the setup is placed with the length perpendicular to the Earth’s gravitational field, natural convection does not play any role and, due to the lack of a temperature gradient along the height, Soret effects will also not play any role. Next to this, also, the temperature dependence of the dielectric constant and electro-osmotic vortices is rather limited, compared to the effect of Joule heating and charge regulation. Meanwhile, the introduction of protons and hydroxides will have an influence on the current efficiency; the pH is expected to be rather constant within the depletion zone.

### 6.8 Effect of Zeta Potential and Zeta Potential Corrections

**Figure 15:**
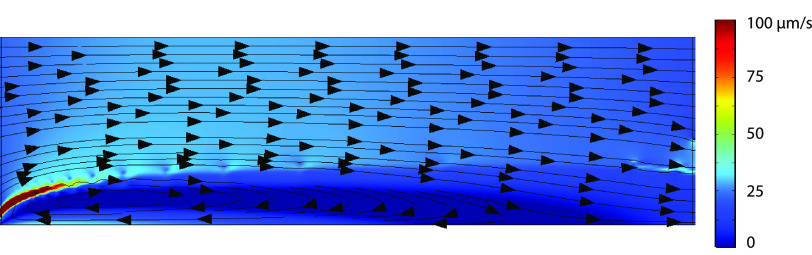
Velocity in the second domain for nonisothermal charge regulation simulation, which includes the zeta potential from the CR model at an applied potential of 60 V.

Up to this section, we have kept the zeta potential at a fixed value of −70 mV, to focus on the effect of charge regulation on the surface charge density of the porous medium. However, in reality, the zeta potential will increase as the salt concentration decreases. Because of the decreasing concentration along the length of the cross-flow setup, the zeta potential and the magnitude of the electro-osmotic flow component each increase along the length of the setup, assuming that the CR model holds; this leads to values around −190 mV. Since the fluid is assumed to be unable to pass the horizontal boundaries, the EOF leads to pressure buildup at the bottom boundary. Because of ion depletion, the zeta potential and consequently the magnitude of the EOF increases along the length. This results eventually into a recirculation pattern inside the depleted layer, which can be seen in Figure 15.

This mixing zone mixes the depleted salt stream with the enriched stream, which has a negative impact on the salt content of the depleted water and therefore requires larger applied potentials, relative to the constant zeta potential model, to reach equivalent desalination degrees, as can be seen later in this paper (in Figure 18).

**Figure 16:**
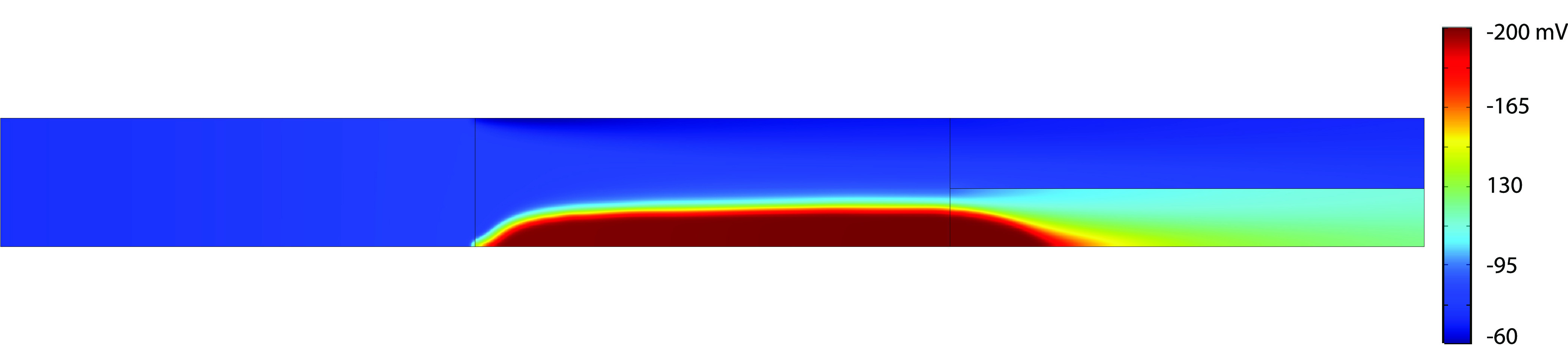
Local zeta potentials at an applied potential of 30 V.

**Figure 17:**
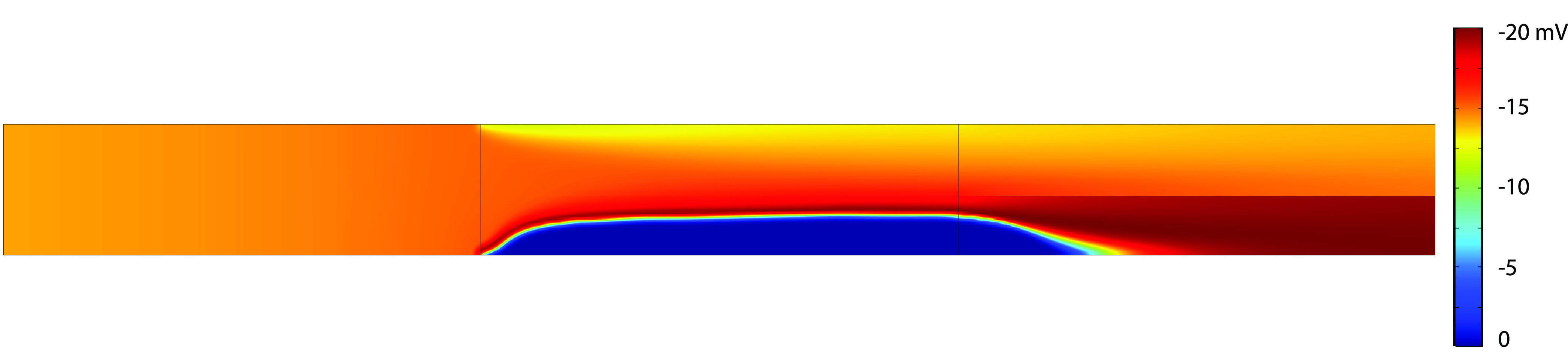
Local effective zeta potentials (zeta potential multiplied by correction factor) corrected for double-layer overlap at an applied potential of 30 V.

In the depleted regions, very high zeta potentials are obtained. However, CR models are typically not verified under these extremely low salt concentrations. Compounding this effect, for highly depleted regions using the Helmholtz–Schmoluchowski model for electro-osmotic flow will definitely break down in the depleted region, since the Debye length is no longer thin, compared to the porous medium, as discussed in section 3. To correct the electro-osmotic flow for the double layer thickness, relative to the pore size of the porous media, a correction factor has been proposed in the past as a function of the double layer thickness by Coelho et al.^33^ Its most simple form can be written as follows:
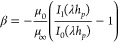
23where  = 0.186 for a porous medium composed of uncharged spherical particles. *I*_1_ and *I*_0_ are the corresponding first-order modified Bessel functions, κ is the inverse Debye length, and *h*_p_ is the average pore size in the porous medium. The effect of this correction factor on the zeta potential can be seen in Figures 16 and 17. From these figures, it can be concluded that, even though the zeta potential calculated based on the CR model obtains very high values, the effective electro-osmotic flow is close to zero, for high applied potentials, due to the correction factor.

**Figure 18:**
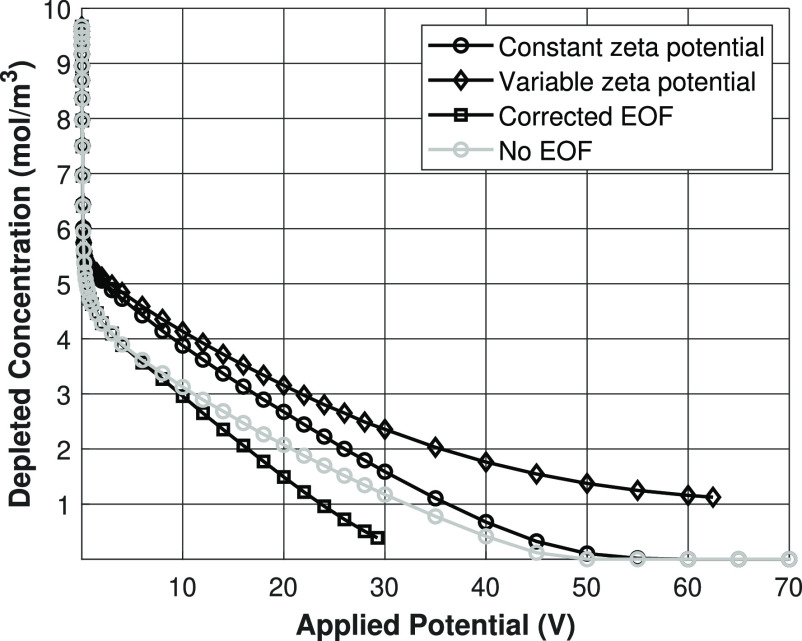
Depleted concentration versus the applied potential for the different EOF models: Constant zeta potential (base case), variable zeta potential, double-layer corrected EOF, and no EOF. Lines are present for visualization purposes only.

To show the effect of the assumed zeta potential model on the performance of the shock ED process, finally, the concentration is plotted against the applied potential once more for the previously discussed zeta potential models and a model without EOF (see Figure 18). In this figure, it can be seen that the variable zeta potential requires far more driving force to remove salt from the depleted stream, compared to the constant zeta potential model case. This is due to the recirculation that was shown earlier in this section. Interestingly, the double-layer corrected EOF and no EOF simulation results show quite some similarities, which is also expected, based on the described effect of double-layer overlap on the EOF in the work of Coelho et al.^33^ To visualize the effect of the double-layer correction factor, the zeta potential multiplied by the correction factor, called the “effective zeta potential”, is shown in Figure 17. This figure shows that the effective zeta potential inside the depleted layer is effectively zero, which results in almost no EOF in the depleted zone. Compared to the constant potential simulation, the corrected and no EOF simulations show somewhat improved performance, especially between a depleted outlet concentration of 5 and 1 mol/m^3^. It is thought this is due to the phenomenon that electro-osmotic flow flowing from the top to the bottom will introduce some concentrate back into the depleted zone, which counteracts the desalination process, to some extent. However, the EOF definitely plays some role in transporting the ions by convection toward the depletion zone, since, at concentrations below 1 mol/m^3^, the difference between the constant zeta potential model and the no EOF model diminishes. In addition, the desalination in the corrected EOF model, relative to the no EOF model, proceeds at somewhat lower potentials. However, the effect of the electro-osmotic flow is quite limited, compared to the effect of the surface charge of the porous medium.

While it is interesting to see the influence of different ways to implement the EOF and zeta potentials, when put into perspective, the influence of the EOF on the desalination performance is quite limited, compared to the main effects studied in this work, which justifies keeping the zeta potential constant in our current and further studies.

### 6.9 Electric Cost Evaluation

**Figure 19:**
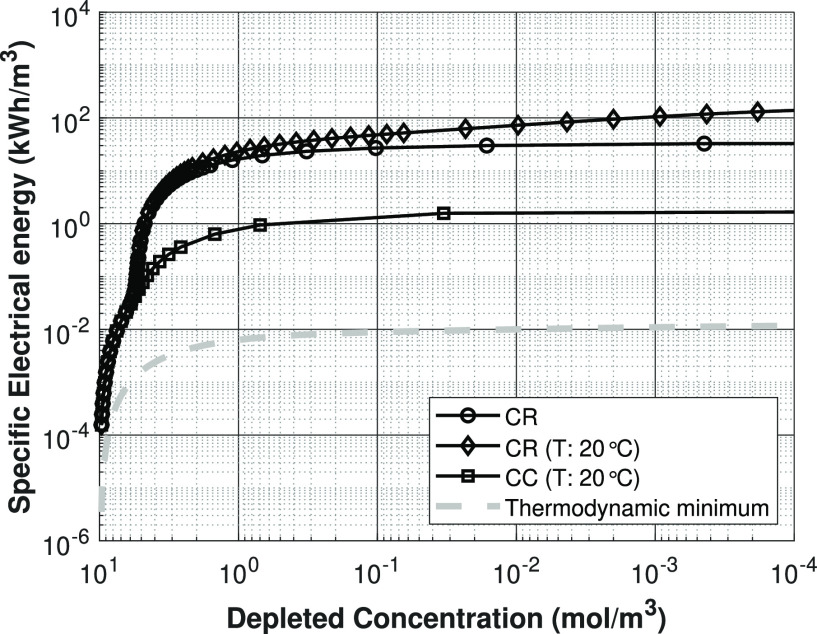
Electrical energy cost per m^3^ desalinated water for the constant charge, the charge regulation cases and the thermodynamic minimum based on a Gibbs free energy calculation. Lines are present for visualization purposes only.

To put into perspective how charge-regulation effects can alter the energy costs of a shock ED process, the electrical energy costs for a single-stage shock ED process are calculated as a function of the depleted outlet concentration from our simulation results (see Figure 19). This is a rough estimation of the costs of the process, considering the simplifying assumptions involved in the various shock ED models used in this work. However, it gives a clear indication of the influence CR-type effects has on the cost of possible shock ED processes. The electric costs for the main simulation cases are calculated by using the relation
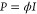
24where *P* is the electrical power, ϕ the applied electric potential (V), and *I* the current (A) needed to produce a cubic meter of depleted water. By subsequently calculating the time needed per cubic meter of depleted water obtained, the energy consumption per cubic meter of depleted water can be calculated.

The theoretical minimum electric energy consumption is calculated based on the ideal Gibbs free energy of mixing of both the inlet and outlet streams:
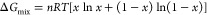
25where Δ*G*_mix_ is the Gibbs free energy of mixing, *R* the universal gas constant, *T* the temperature, and *x* the fraction of salt present in the stream.

By calculating the difference between the inlet Gibbs free energy and the outlet Gibbs free energy, the energy required for the separation of salt can be estimated with the relation
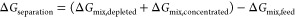
26

The general shape of all the electric cost curves is identical (see Figure 19). Initially, the energy cost is fairly low for barely desalinated salt solutions. From there up until 90% desalination, the electrical costs rise very quickly. Past 90% desalination, the further increase in electrical energy costs with salt concentration is only marginal. The CC density simulation results already indicate that the energy costs will be ∼1000 times higher, compared to the theoretical minimum and logically because the charge regulation requires a higher applied potential, the more realistic costs are even 50 times higher compared to the constant charge case. For conventional electrodialysis, the energy cost to desalinate 1 g/L NaCl solution (17 mol/m^3^) up to 80% is ∼0.08 kWh/m^3^ fresh water,^34^ which is about an order of magnitude lower, compared to the CC simulation, when a slightly higher feed salt concentration is desalinated. Thus, comparing the shock ED electrical energy costs highlights that it will be challenging to implement shock ED cost-effectively in an industrial process, especially since conventional electrodialysis is more efficient, in terms of electrical costs. Our conclusion about the electrical efficiency is also consistent with a recent review by Alkhandra et al.,^35^ where it was shown that the shock ED process had very low efficiency, relative to other electrically driven desalination processes. In addition, in our energy analysis, we also neglected the energy required for pumping the liquid through the porous medium, which is higher, compared to conventional electrodialysis, because of the higher hydraulic resistance of porous media. This highlights the current challenges facing shock electrodialysis as an electrical desalination technique, since the advantage of needing fewer filtration pretreatment steps must outweigh the higher electrical and pumping costs of shock ED, relative to conventional electrodialysis.

## 7 Conclusions

In this work, we have extended the existing theoretical model of shock ED by investigating the shock ED process with a charge-regulating (CR) porous silica medium including Joule heating and associated temperature effects on the ion transport. First, we showed that a CR porous medium rapidly loses its surface charge with decreasing concentration, which is partially compensated for by Joule heating. As a consequence of this, far higher applied electrical potentials are needed to desalinate the solution in a CR medium, compared to a porous medium with a constant surface charge density.

Next, we showed that even though the electro-osmotic flow could become more intense for CR media, due to the higher applied potentials, the relative fraction of depleted water is not substantially enhanced until the solution is completely depleted. This implies that the enhancement of the depleted water recovery is possibly not achieved in the shock ED process, under realistic operating conditions.

When comparing our results with the results obtained by Schlumpberger et al^11^ and Tian et al.,^14^ we observed that, for the degree of desalination versus the dimensionless current figure, the simulations that did not consider protons and hydroxides completely coincided with each other.

We also found that the influence of electro-osmotic flow (EOF) on the desalination performance was limited in the cases we considered. This can mean that more precise modeling of EOF in the porous medium is possibly of less importance for Shock ED applications. Finally, we observed that the electrical energy costs of shock ED to desalinate brackish water, containing only 10 mol/m^3^ of sodium chloride, for our currently modeled setup geometry, is far higher than the costs for conventional electrodialysis for all presented modeling cases. This highlights the challenges involved in implementing shock ED on an industrial scale to treat brackish waters cost-effectively.

Throughout this work, it was shown that the charge density is the most important parameter that determines the performance of the shock ED process. Therefore, using silica as a porous medium may be a less-than-ideal choice, since its charge is greatly diminished by the decreasing salt concentration. Therefore, modifying the silica surface or choosing a porous medium material that has a higher charge density or with a reduced dependence of charge density on salt concentration can reduce the energy consumption for desalination when using shock ED to some extent, which is illustrated in Figure S2 in the Supporting Information. In this work, we considered the case of nonadsorbing ions in a shock ED process. However, it would be interesting to extend this modeling study to a feed with multivalent cations, since they are known to adsorb onto silica,^20,36^ as well as mixtures. This could have large implications on the charge density of the porous medium, which is relevant to theoretically describe the behavior of shock ED process with complex feed streams, such as the recent experimental work of Alkhadra et al.^6^

## Supporting Information Available

Evaluation of the influence of other temperature effects (Figure S1); evaluation of the influence of electro-osmotic vortices (Figure S2); evaluation of the effect of fixed pH assumption (Figure S3); evaluation of the constant charge models with different charge densities (Figure S4) (PDF)
