# Supplementary material for: Influence of Charge Regulation on the Performance of Shock Electrodialysis
Source: Ind Eng Chem Res. 2023 Feb 7;62(7):3294–306. doi: 10.1021/acs.iecr.2c03874 (PMC9951225; doi:10.1021/acs.iecr.2c03874)
Supplement: Supplementary file 1 — ie2c03874_si_001.pdf [file ie2c03874_si_001.pdf]

Influence of Charge Regulation on the Performance of Shock Electrodialysis

Harm T.M. Wiegerinck, Reinder Kersten, and Jeffery A. Wood*

Soft Matter, Fluidics and Interfaces, MESA+ Institute for Nanotechnology, University of Twente, 7500AE Enschede, The Netherlands

E-mail: j.a.wood@utwente.nl

Supporting Information Available

Contents

Supporting Information Available **S1**

S1 Detailed information on other temperature effects **S2**

S1.1 Buoyancy effect S2

S1.2 Soret Effect S4

S1.3 Effect of Temperature on the Dielectric Constant S4

S2 Electroosmotic vortices in porous media **S5**

S3 Effect of fixed pH assumption **S7**

S4 Charge Density Comparison **S8**

References **S9**

S1 Detailed information on other temperature effects

S1.1 Buoyancy effect

Free convection arises when are density differences along the Earth's gravitational field. The density of water decreases with temperature and therefore temperature gradients could lead to natural convection. Since it is established in section 6.1 that there is only a large temperature gradient along the length of the setup. Only when the setup is positioned as indicated on the right of Figure S1 this could potentially lead to natural convection.

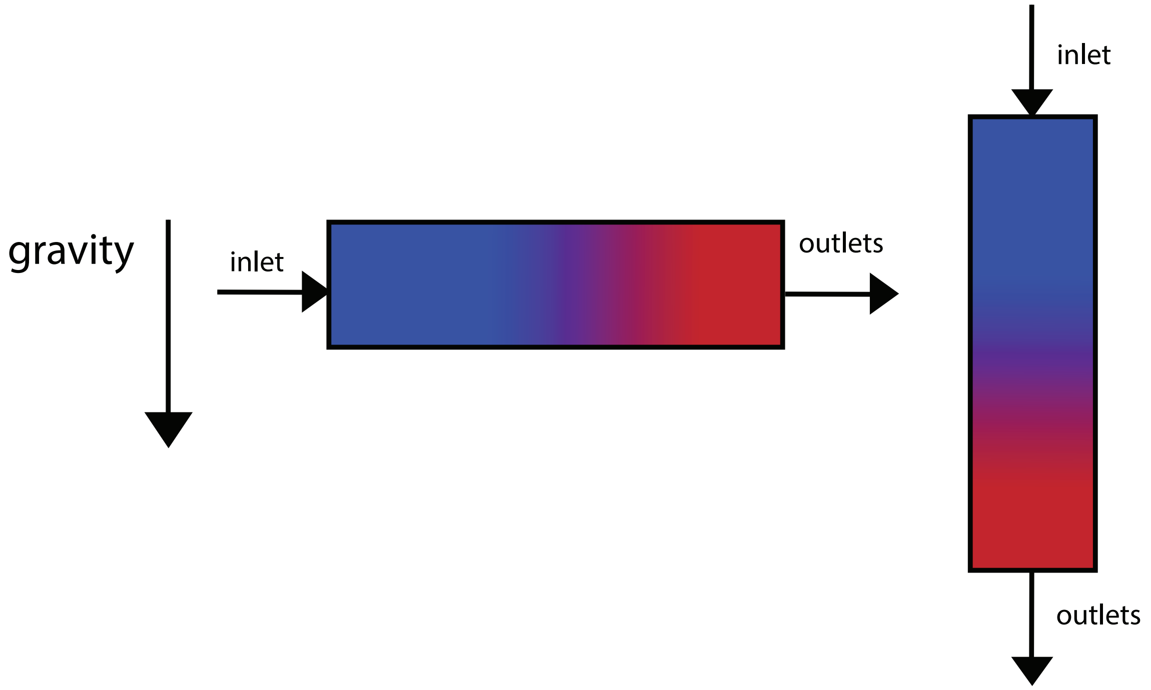

Figure S1: 2 Different orientations of the shock ED setup with respect to gravity. Right the normal orientation of continuous shock ED cell. Left the orientation where natural convection can have a large influence on the process. Colors indicate the local temperature where red and blue indicate the hot and cold fluid respectively.

To evaluate the influence of the free convection in that particular case with respect to the forced convective fluid flow, the Richardson number is evaluated, which gives the ratio between forced convection and natural convection as ratio between the Grashof and the square Reynolds numbers

$$Ri = \frac{Gr}{Re^2} \tag{1}$$

The thermal Grashof number is defined as:

$$Gr = \frac{g\beta \Delta T D^3}{\nu} \tag{2}$$

with g acceleration due to gravity,β the thermal expansion coefficient of water and ν the kinematic viscosity of water. By filling in the approximate overall temperature difference between the inlet and outlet of the non-isothermal charge regulation model at 90 V, the Grashof number is approximately equal to 930.

The Reynolds number is given by:

$$Re = \frac{\rho u D}{\mu} \tag{3}$$

Under the same circumstances as for the previous calculation of the Grashof number, the Reynolds number is equal to approximately 0.1. This results in a Richardson number of 93000.

This means that when the shock ED is oriented as indicated by the right of Figure S1, the free convection will probably completely dominate the pressure induced forced convection. Therefore, with a temperature difference the water should be pumped through at a higher pressure to overcome the natural convective flows, which will increase the salinity of the depleted stream at similar applied potentials. Therefore, in practice it is highly undesirable to allow natural convection to occur in the shock ED process. However, based on the geometry, such a setup will most likely be positioned like the left of Figure S1 and in that case free convection will not play such a distinct role in the hydrodynamics.

S1.2 Soret Effect

. The Soret effect, also known as thermophoresis, results in enhanced ion transport, when there are considerable temperature gradients present in a system. The Soret flux for the cations can be described by:

$$J_{\text{Soret}} = D_+ c_+ \frac{Q^*}{RT^2} \nabla T \tag{4}$$

where the Q^* is the heat of transport which is for Na^+ equal to 3.46 kJ/mol as given by Würger.[1]

By comparing the flux expression to the total flux towards the electrode boundary of the non-isothermal charge regulation model, it was calculated that the Soret flux will be at least 1000 times smaller, outside the depletion zone compared to the total ion flux. While inside the depletion zone this ratio was even larger and the Soret flux would be approximately a million times smaller compared to the total flux. This can be attributed to the lack of a large temperature gradient, as discussed in previous section, that drives the ions. Therefore it is probably safe to conclude that the effect of thermophoresis will be negligible in shock ED simulations.

S1.3 Effect of Temperature on the Dielectric Constant

The effect of temperature on the relative dielectric constant of water is discussed. Since in our description of shock ED the electroneutrality condition is used, the dielectric constant only affects the Debye length (Debye parameter), which in turn affects the surface charge density of the porous medium. To describe the temperature dependence, the following empirically determined equation was used.[2]

$$\epsilon_r = 87.740 - 0.4008T + 9.398 \cdot 10^{-4} T^2 - 1.410 \cdot 10^{-6} T^3 \tag{5}$$

where ϵ_r is the relative dielectric constant and T is the temperature in degree C. Note that this empirical relation is strictly only valid for water without any salt. However, the dielectric constant is not very sensitive to the salt concentrations and only for water such as sea water, it will change the dielectric constant[3] significantly. Since in our work we only studied brackish salt concentrations (10 mol/m^3), the current description of the dielectric constant change with temperature will suffice.

Under the conditions used in this simulation, it turned out that the average dielectric constant in the depleted zone changed from an average relative dielectric constant of 80 to approximately 65 at at 293 K and 343 K respectively. However at applied potentials where the depleted salt outlet concentration is still significant, the average relative dielectric constant only changed from 80 to 76.

Table S1: The effect of the temperature on the dielectric constant and an evaluation of the effect of the changing dielectric constant on the depleted outlet concentration

Applied Potential	Dielectric constant	Concentration (mol/m^{-3})	
		constant ϵ_r	TD ϵ_r
35	78	1.11	1.10
45	77	0.32	0.31
55	76	1.6x10^{-2}	2.39x10^{-4}

As a consequence of this there was barely any change in desalination performance by including the temperature dependence of the dielectric constant, which can be observed in Table S1. However, for desalinating higher concentrations of salt or when using the full Poisson-Nernst-Planck model as was done by Tian et al,[4] the effect of including the temperature dependence of the dielectric constant could have a larger impact.

S2 Electroosmotic vortices in porous media

Recently it was found that electroosmotic vortices may form in porous materials during shock ED, due to different local pressures within the connected pores. This could enhance the ion

transport in shock ED.[5] However, in the continuum model presented here, the electroosmotic flow is largely counteracted by the resulting pressure gradient at the bottom wall, which means that net ion transport was not strongly affected by convective flows.

To investigate the effect of electroosmotic vortices with a continuum model, we included an enhanced diffusion coefficient description based on Taylor-Aris kind of dispersion as described by Licon Bernal et al.[6]

The enhanced diffusion coefficient due to electroosmotic vortices is described by

$$D_{EOV} = D\left(1 + \frac{2}{105}\frac{h^2 u_{EO}}{D^2}\right) \tag{6}$$

where h is originally the microchannel size and here it is assumed to be equal to the average pore size (h_p), u_{EO} is the electroosmotic velocity (in the y-direction)

To ensure the effect of electroosmotic vortices is only applied in the direction of the electroosmotic flow, we added the diffusion coefficients as a so-called diagonal matrix, with the usual diffusion coefficient in x-direction and the enhanced diffusion coefficient in the y-direction.

When evaluating the ratio of the enhanced diffusion coefficient over the usual diffusion coefficient, calculated by takeing the average over the bottom electrode boundary, It can be seen that at these potentials, the enhancement is about 13 to 16 percent compared to the temperature corrected diffusion coefficient (see Table S2). While this does not seem to be a very large difference, the relative difference in the outlet concentrations ranges from about 15 to 70 percent at a potential of 35 and 55 V respectively, when comparing the electroosmotic vortices case with the CR case. This difference is probably due to the fact that for increasing potential, the depletion layer grows in size, which leads to a larger region where the diffusion coefficient is higher compared to the bulk value. However, one should realize that although the relative differences are quite large, the absolute numbers are at that point already quite small.

To conclude, when the effect of electroosmotic vortices on the ion transport is included, the ion transport is slightly enhanced, which means that it could be a valuable addition to describe the shock ED process better. When put in perspective the influence of charge-regulation (variable charge density) was much larger relatively. However, since the correlation used was developed for electroosmotic vortices enhancing diffusion in a single microchannel, it is possible that these effects could play a larger role.

Table S2: Comparison between the depleted concentrations without the enhanced diffusion coefficient due to electroosmotic vortices (EOV) and the model that includes this effect, for 3 different applied potentials.

Applied Potential	D_{Na^+EOV}/D_{Na^+}	Concentration (mol/m^3)	
		No EOV	with EOV
35	1.13	1.1	0.94
45	1.14	0.32	0.21
55	1.16	$1.6\cdot 10^{-2}$	$5.0 \cdot 10^{-3}$

This analysis shows that the effect on ion transport of electroosmotic vortices might be limited in continuum simulations. In a more realistic shock ED setup that includes transitions from free fluid to the porous medium (region 1 to 2 in Figure 3) and vice versa (region 2 to 3 in Figure 3), as has been done by Tian et al.[4,7] In this work, it was shown that electroosmotic flows near these transitions also result large electroosmotic vortices, which partially mixes the feed salt solution with the depletion layer. These vortices will results in an increase the energy consumption of Shock ED processes compared to the energy consumption considered in our simulation cases.

S3 Effect of fixed pH assumption

Throughout this work, we neglected the effect of the formation of protons and hydroxides due to the water equilibrium or electrode reactions. Naturally, the current efficiency will drop due to the presence of these ions, which seems to lead to deviations between experimental and our simulation results. However, since in reality the protons will be transported through

the ion exchange membrane, it is expected that within the depleted layer the pH will remain rather constant, which is also suggested by the 2D figures presented in the work of Tian et al.[4] This means that due to the lower current efficiency the driving force needs to be somewhat higher compared to the results presented in this work but the charge of the porous medium is not altered too much in the simulations. Therefore it is expected that this assumption will not have as much effect on the theoretical performance as the charge regulation and Joule heating have.

S4 Charge Density Comparison

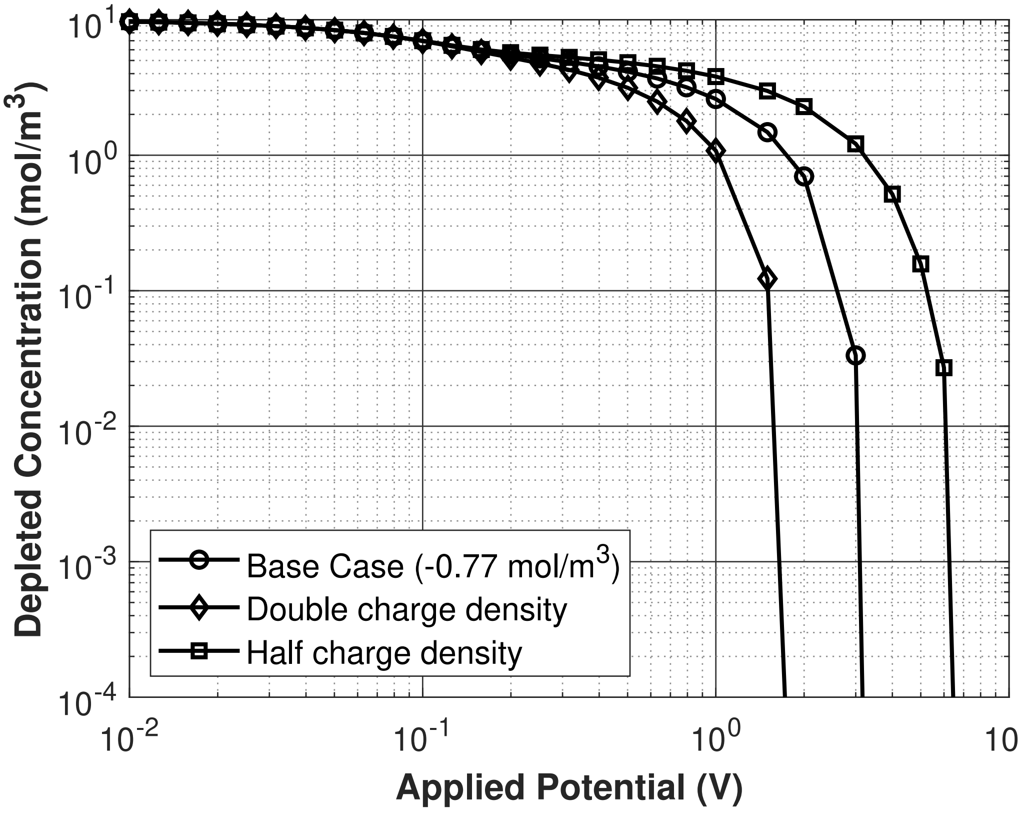

Figure S2: The depleted concentration versus the applied potential for 3 different porous medium constant charge density cases.

In Figure S2, the desalination performance for 3 constant charge models with different charge densities is presented. In this figure it can be seen that, especially at the highest applied potentials, for a certain depleted concentration, the charge density determines proportionally the potential that needs to be applied to reach a certain degree of desalination. This shows the importance of having a porous medium that has higher charge densities in similar salt concentrations compared to silica to improve the efficiency of the shock ED process to some extent.

References

(1) Würger, A. Transport in charged colloids driven by thermoelectricity. *Physical Review Letters* **2008**, *101*, 5–8.

(2) Malmberg, C.; Maryott, A. Dielectric constant of water from 0 to 100 C. *Journal of Research of the National Bureau of Standards* **1956**, *56*, 1.

(3) Stogryn, A. Equations for Calculating the Dielectric Constant of Saline Water (Correspondence). *IEEE Transactions on Microwave Theory and Techniques* **1971**, *19*, 733–736.

(4) Tian, H.; Alkhadra, M. A.; Bazant, M. Z. Theory of shock electrodialysis II: Mechanisms of selective ion removal. *Journal of Colloid and Interface Science* **2021**, *589*, 616–621.

(5) Mirzadeh, M.; Zhou, T.; Amooie, M. A.; Fraggedakis, D.; Ferguson, T. R.; Bazant, M. Z. Vortices of Electro-osmotic Flow in Heterogeneous Porous Media. *Physical Review Fluids* **2020**, *5*, 103701.

(6) Licon Bernal, E. E.; Kovalchuk, V. I.; Zholkovskiy, E. K.; Yaroshchuk, A. Hydrodynamic dispersion in long microchannels under conditions of electroosmotic circulation: II. Electrolytes. *Microfluidics and Nanofluidics* **2016**, *20*, 58.

(7) Tian, H.; Alkhadra, M. A.; Bazant, M. Z. Theory of shock electrodialysis I: Water dissociation and electrosmotic vortices. *Journal of Colloid and Interface Science* **2021**, *589*, 605–615.
